# The Diagnostic Accuracy of Ultrasound in Assessment of Myometrial Invasion in Endometrial Cancer: Subjective Assessment versus Objective Techniques

**DOI:** 10.1155/2017/1318203

**Published:** 2017-07-24

**Authors:** Filip Frühauf, Michal Zikan, Ivana Semeradova, Pavel Dundr, Kristyna Nemejcova, Ladislav Dusek, David Cibula, Daniela Fischerova

**Affiliations:** ^1^Gynecologic Oncology Centre, Department of Obstetrics and Gynecology, First Faculty of Medicine, Charles University, General University Hospital, Prague, Czech Republic; ^2^Department of Pathology, First Faculty of Medicine, Charles University, General University Hospital, Prague, Czech Republic; ^3^Institute for Biostatistics and Analyses, Faculty of Medicine, Masaryk University, Brno, Czech Republic

## Abstract

The aim of this study was to assess the diagnostic accuracy of subjective ultrasound evaluation of myometrial invasion of endometrial cancer and to compare its accuracy to objective methods. All consecutive patients with histologically proven endometrial cancer, who underwent ultrasound evaluation followed by surgical staging between January 2009 and December 2011, were prospectively enrolled. Myometrial invasion was evaluated by subjective assessment using ultrasound (<50% or ≥50%) and calculated as deepest invasion/normal myometrium ratio (Gordon's ratio) and as tumor/uterine anteroposterior diameter ratio (Karlsson's ratio). Histological assessment from hysterectomy was considered the gold standard. Altogether 210 patients were prospectively included. Subjective assessment and two objective ratios were found to be statistically significant predictors of the myometrial invasion (AUC = 0.65, *p* value < 0.001). Subjective assessment was confirmed as the most reliable method to assess myometrial invasion (79.3% sensitivity, 73.2% specificity, and 75.7% overall accuracy). Deepest invasion/normal myometrium (Gordon's) ratio (cut-off 0.5) reached 69.6% sensitivity, 65.9% specificity, and 67.3% overall accuracy. Tumor/uterine anteroposterior diameter (Karlsson's) ratio with the same cut-off reached 56.3% sensitivity, 76.4% specificity, and 68.1% overall accuracy. The subjective ultrasound evaluation of myometrial invasion performed better than objective methods in nearly all measures but showed statistically significantly better outcomes only in case of sensitivity.

## 1. Introduction

Endometrial cancer (EC) is the most common gynecological neoplasm and the fourth most frequent site of malignancy in females in Europe and North America. Its incidence is highest in economically developed countries exceeding a rate of 14.7 per 100000 women. The mortality only reaches a rate of 2.3 per 100000 women, due to detection at early stages because of early clinical manifestation mostly as abnormal uterine bleeding [1].

The generally favorable prognosis can be influenced by known prognostic markers, including the age of the patient, histological subtype, grade of tumor, tumor size, extent of myometrial invasion, cervical stromal invasion, and spread to the lymph nodes [2, 3]. The “high risk” subgroup of endometrial carcinomas is characterized by the presence of at least one of following independent prognostic factors: poor differentiation (Grade 3), nonendometrioid histological subtype (serous or clear cell adenocarcinoma, carcinosarcoma, etc.), deep myometrial invasion (≥50% of myometrial width), and/or the presence of cervical stromal invasion. It is mostly accepted that these “high risk” patients may benefit from more radical surgery, namely, regional lymph nodes removal. By contrast, in women with “low risk” subgroup of EC, who are not rarely compromised by age, obesity, hypertension, and diabetes, inadequately radical surgery can lead to increased morbidity caused by induced complications without any benefit on survival [4, 5].

The imaging routine in preoperative assessment of EC differs amongst the gynecologists; however it was shown that the accuracy of high-end ultrasound (US) in experienced hands is comparable to that of magnetic resonance imaging (MRI) [6–8]. Furthermore, there are several factors which favor US such as costs, availability, and applicability to all patients. According to recent European guidelines (ESGO/ESMO/ESTRO) the preoperative work-up should include pelvic examination, histopathological assessment of endometrial biopsy (histological subtype and grade), and transvaginal or transrectal US [9].

Subjective evaluation of myometrial invasion in the hands of experienced examiners reaches sensitivities from 61 to 93% and specificities from 71% to 92% [6, 7, 10–14]. In addition, the sensitivities and specificities of subjective assessment of cervical stromal invasion range from 25% to 93% and from 85% to 99%, respectively [6–8, 10–12]. Several studies evaluated objective measurement techniques for US to stage the disease, especially the myometrial invasion [12, 14–16]. One of the traditional approaches was that proposed by Gordon et al. using the ratio between the distance of the maximum tumor invasion and total width of myometrium [17]. The sensitivity of US in detecting the level of myometrial invasion based on this calculation was 76% while the specificity was 75% in a group of 25 women. The tumor/uterine anteroposterior (AP) diameter ratio suggested by Karlsson et al. with cut-off >0.5 denoting objectively deep myometrial invasion reached a sensitivity of 79% and a specificity of 100% in a study involving 30 women [18]. In 2015 Alcázar et al. published a systematic review and meta-analysis based on preoperative detection of deep myometrial invasion comparing subjective assessment and Gordon's and Karlsson's ratios [19]. Between 1989 and 2014 only 24 studies were identified using either subjective assessment or Gordon's or Karlsson's ratio, but none of these studies compared the three methods altogether in one cohort of patients and significant heterogeneity was found for sensitivity and specificity. Therefore, the aim of this prospective study was to assess the diagnostic accuracy of subjective assessment of myometrial invasion in endometrial cancer and to compare its accuracy to objective measurements using two traditional sonographic parameters (Gordon's and Karlsson's ratios) in the same population of patients.

## 2. Methods

All consecutive patients with histologically proven endometrial cancer who underwent US examination followed by surgical staging between January 2009 and December 2011 at Gynecological Oncology Centre were prospectively enrolled into the study. Magnetic resonance imaging was not used as the routine primary imaging method in our institution.

The preoperative biopsy was obtained by hysteroscopy or D&C and tumor grade and histological subtype were recorded. Histology from endocervical curettage was registered separately, if available.

The standardized study protocol containing all investigated sonographic parameters was defined before the beginning of the study. Each patient was examined by one of experienced sonographers (D. F., M. Z., and I. S.) within one month before staging surgery. The final surgical procedure included extrafascial abdominal or laparoscopic hysterectomy both with salpingo-oophorectomy. Radical hysterectomy was only performed if cervical stroma was involved in US scan. Based on preoperative histological grade, subtype, and subjective US assessment of local stage, lymphadenectomy was omitted in “low risk” subgroup (endometrioid Grade 1 or 2 and superficial myometrial invasion < 50%), while all other patients suitable for radical surgery were referred for a systematic pelvic and para-aortic lymph nodes dissection.

**Figure 1:**
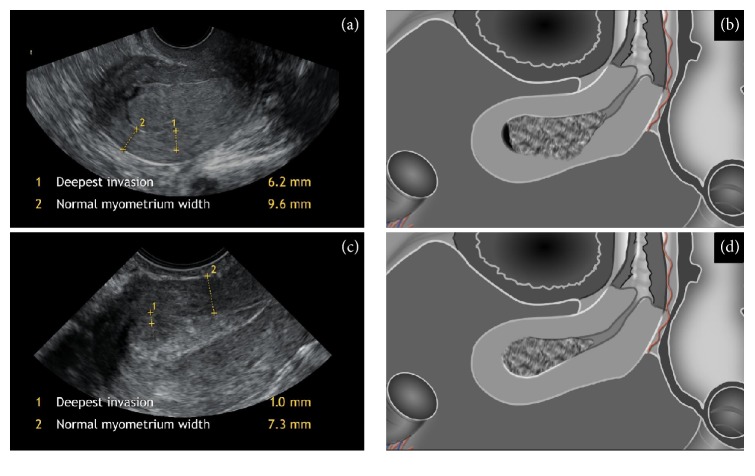
Ultrasound images and schematic diagrams showing* deepest invasion, *the largest distance in any plane between endometrium-myometrium junction and maximum tumor depth, and corresponding* normal myometrium *assessed as the myometrial width aside of the deepest tumor invasion without fibroids; (a-b) deepest invasion/normal myometrium ratio ≥ 0.5 reflecting the deep invasion, histologically proven FIGO stage IB; (c-d) deepest invasion/normal myometrium ratio < 0.5 indicating superficial invasion, histologically proven FIGO IA.

**Figure 2:**
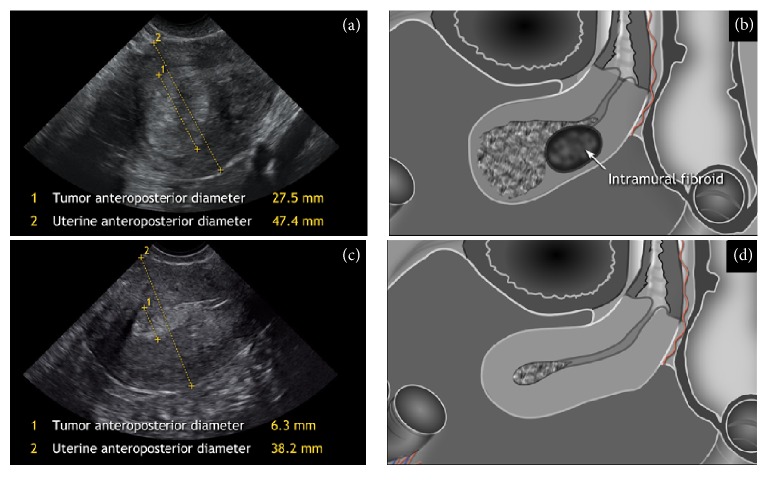
Ultrasound images and schematic diagrams showing* tumor anteroposterior (AP) diameter, *maximum width of the tumor in sagittal plane, and* uterine anteroposterior diameter, *AP diameter of the uterus measured at the same place; (a-b) tumor/uterine AP ratio ≥ 0.5 reflecting the deep invasion, histologically proven FIGO stage IB; (c-d) tumor/uterine AP ratio < 0.5 indicating superficial invasion, histologically proven FIGO stage IA.

The US equipment used in this study was a Voluson E8 (GE Medical Systems, Zipf, Austria) with a RIC5-9 transducer and multifrequency endovaginal probe (5–9 Hz). The US scans were performed in lithotomy position after emptying the bladder. Transvaginally, the whole uterus was observed in sagittal section from one uterine lateral border to the contralateral one and in transversal section from the cervix to the fundus as published previously [35]. The tumor was evaluated on 2D gray-scale in B-mode and its characteristics were described using IETA terminology [36]. Doppler was used, if necessary to establish tumor borders on the basis of vascular pattern or to identify the feeding vessel of the polyp as the point of expected deepest invasion. The US variables that were evaluated during real-time two-dimensional examination included data on uterus and tumor size in three perpendicular diameters, deepest myometrial invasion, minimal tumor-free margin and corresponding normal myometrium width, presence, location, and number of fibroids. Deepest myometrial invasion was measured as the distance between endometrium-myometrium junction and maximum tumor depth. Minimal tumor-free margin was assessed as the smallest distance between tumor and serosa. Corresponding normal myometrium was measured as total myometrial width aside from the tumor deepest invasion without fibroids (Figure 1). In sagittal plane maximal AP diameter of the uterus and maximal AP diameter of the tumor were registered (Figure 2). Static images with all measurements and video-clips with or without Power Doppler were collected for each patient and examination protocols were recorded immediately after image acquisition.

Myometrial invasion was evaluated by subjective assessment (<50% or ≥50%) and objectively calculated as deepest invasion/normal myometrium width ratio (a quota ≥ 0.5 reflecting the deep invasion) introduced by Gordon et al. [17] (Figure 1) or as tumor/uterine AP ratio (accordingly ≥ 0.5 indicating the deep invasion) formerly investigated by Karlsson et al. [18] (Figure 2). The selected cut-off limit for the extent of myometrial invasion (0.5) followed FIGO staging classification [37].

To complete US local staging of EC, cervical infiltration was evaluated by subjective assessment as “absence” or “presence of stromal invasion” and the findings were recorded. The dynamic test was used to display the sliding effect of the tumor mass along the cervical wall and thus to distinguish the simple protrusion from the actual invasion to the cervical stroma. Another clue was the pronounced stromal vascularization below the level of uterine arteries in isthmus which accompanies the cervical stromal invasion [35].

Surgical specimens were examined by dedicated pathologists with substantial experience in gynecologic oncology using a predetermined protocol regarding: histological subtype, grade, lymphovascular invasion, tumor size in three diameters, presence, location and number of fibroids, depth of myometrial invasion, minimal tumor-free myometrium, corresponding intact myometrial width, and presence of cervical stromal invasion. Endometrioid adenocarcinomas were divided into 3 grades (Grade 1 = well differentiated, Grade 2 = moderately differentiated, and Grade 3 = poorly differentiated). The tumor classification followed the recommendation of the World Health Organization (WHO) and the criteria of the International Union Against Cancer (TNM Classification of malignant tumors) were used for pathological tumor staging [38]. The International Federation of Gynecology and Obstetrics (FIGO 2009) criteria were applied for clinical staging [37]. The “gold standard” was based on final histology of the specimen obtained by hysterectomy.

*Statistical Analysis*. Data were reported as median estimate supported by 5th and 95th percentile range for continuous variables and absolute and relative frequencies for categorical variables. ROC (Receiver Operating Characteristics) curves with 95% confidence intervals were calculated for both objective US measurement techniques and for subjective assessment. Comparison of the predictive power of subjective evaluation and objective methods in assessment of myometrial invasion and comparison of subjective assessment of myometrial and cervical stromal invasion were based on chi-square test. The result of postoperative histology was taken as reference outcome. AUC (area under curves) values for these three diagnostic modalities were compared using an algorithm published in Hanley and McNeil (1982) [39]. Sensitivity, specificity, positive predictive value (PPV), negative predictive value (NPV), and overall accuracy were calculated. Statistical analysis was carried out using SPSS software version 20.0.0 (IBM Corporation, 2011) and MedCalc 12.3.0.0 (MedCalc Software 1993–2012); *p* values < 0.05 were accepted as the boundary for statistical significance [40].

## 3. Results

Two hundred and ten patients were prospectively enrolled into the study, while only one patient in study period was excluded because of missing ultrasound variables. The median age of the study population was 66 years (range 53; 83), 193 patients (92%) were postmenopausal, and median body mass index was 30 kg/m^2^ (range 21; 47). The most frequently encountered histological subtype and grade was endometrioid (88.1%, 185/210) and Grade 1 (47.6%, 100/210), respectively. The patient demographics and tumor characteristics are summarized in Table 1.

The endometrial cancer was diagnosed using dilatation and curettage (D&C) in 110 patients (52.4%). Hysteroscopy with biopsy was performed in 93 patients (44.3%) and in 7 patients (3.3%) the bioptic method was not specified by the referring gynecologists. Ultrasound scan (index test) was considered by examiner to be of good or moderate quality in 173 cases (82.4%). All ultrasound parameters of the study group recorded during the examination are presented in Table 2.

Regarding final surgical procedure, 124 patients (59.0%) underwent open surgery and 86 patients (41.0%) underwent laparoscopic surgery. Radical hysterectomy was performed in 14 out of 25 patients (56.0%) with suspicious cervical stromal involvement on US. Lymphadenectomy was performed in 121 women (57.6%), from which 17 (14.0%) had histologically proven lymph node metastases.

Final histology (reference standard) reported deep myometrial invasion in 87 patients (41.4%). Myometrial invasion was preoperatively underestimated by subjective US assessment in 18 patients (8.6%) and overestimated in 33 out of 210 patients (15.7%). These data corresponded to a PPV of 67.6% and NPV of 83.3%. Accordingly, Gordon's ratio calculating myometrial invasion as deepest invasion/normal myometrium ratio with cut-off 0.5 reached PPV of 56.7% and NPV of 77.1%. Tumor/uterine AP ratio (Karlsson's ratio) with the same cut-off 0.5 showed PPV of 62.8% and NPV of 71.2%. Subjective evaluation reached 79.3% sensitivity, 73.2% specificity, and 75.7% overall accuracy. Gordon's ratio had 69.6% sensitivity, 65.9% specificity, and 67.3% overall accuracy, which was similar to Karlsson's ratio (56.3%, 76.4%, and 68.1%, resp.). The diagnostic performance of subjective assessment and two objective calculations in predicting deep myometrial invasion as well as the statistical comparison of subjective evaluation to objective methods are introduced in Table 3.

Histological examination described the presence of cervical stromal invasion in 37 patients (17.6%). Underestimation by subjective US assessment occurred in 22 cases (10.5%) and overestimation in 10 out of 210 cases (4.8%). These outcomes corresponded to PPV of 60.0% and NPV of 88.1%. The sensitivity of cervical stromal involvement evaluation was significantly lower than that of myometrial invasion assessment (40.5% versus 79.3%, *p* value < 0.001). However, overall accuracy due to higher specificity (94.2% versus 73.2%, *p* value < 0.001) increased significantly (84.8% versus 75.7%, *p* value 0.019) (Table 3). Only 99 out of 210 patients (47.1%) had available results from endocervical curettage. Therefore, the statistical analysis was limited by sample size and the preoperative assessment of cervical invasion by curettage was not significantly predictive in our study.

## 4. Discussion

In this prospective study on 210 women with endometrial cancer for the first time the subjective evaluation and two objective models of the myometrial invasion assessment (Gordon's ratio and Karlsson's ratio) were compared in the same cohort of patients. All three tested approaches were found to be statistically significant predictors of the myometrial invasion, exceeding AUC value of 0.65 and reaching final *p* value < 0.001. Subjective evaluation revealed being partially better than objective parameters in almost all measures of the diagnostic tests, but statistically significantly better outcomes were reached only in case of sensitivity (*p* value 0.023 and *p* value < 0.001, resp.).

The strength of our work was the prospective design, large number of enrolled patients, and the experience of US examiners and pathologists involved as well as strictly standardized reproducible protocol used to evaluate the histological findings (based on FIGO 2009 and generally recognized IETA recommendations). Finally, the US examination was performed as part of preoperative assessment of endometrial cancer, so it was not associated with extra costs or extra burden for patients. Several prospective studies assessed diagnostic performance of subjective US evaluation and/or objective US measurements using Gordon's or Karlsson's ratio in the prediction of myometrial invasion. However, to the best of our knowledge these three approaches have never been applied altogether to the same dataset of patients (Table 4). The results from studies with consecutive cohorts of ≥50 patients and available data on diagnostic performance including sensitivity and specificity are listed in Table 4 [6, 10–16, 20–34].

The limitations of this study arose from the absence of other preoperative imaging methods for comparison to US evaluation, but it was not the concern of our study since there are enough recent publications showing that MRI and US based on subjective expert assessment perform equally well in the evaluation of myometrial and cervical invasion [6–8]. Another limitation is that we did not assess the intra- and interobserver reproducibility of subjective US evaluation. However, Eriksson et al. recently showed in study including 53 cases that US experts had moderate to good interobserver reproducibility in predicting myometrial and cervical stromal invasion. Moreover, gynecologists with no previous experience in US preoperative staging of EC performed equally well regarding assessment of myometrial invasion but had significantly lower diagnostic performance in assessment of cervical stromal invasion [41]. Lastly, we only focused on two-dimensional transvaginal/transrectal US as it is routine recommended approach for staging, but Christensen et al. demonstrated on 110 patients that three-dimensional ultrasound did not have a higher diagnostic performance in local staging of EC and the results did not improve when saline infusion was added [34].

**Figure 3:**
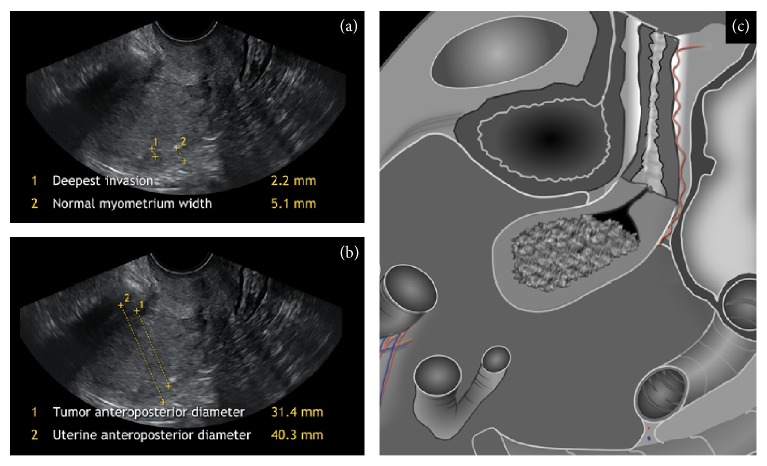
Ultrasound images showing a comparison between two objective methods: (a)* deepest invasion/normal myometrium width ratio* with a quota < 0.5; (b)* tumor/uterine AP ratio* reaching a quota ≥ 0.5 in the same case of large polypoid endometrial carcinoma surrounded by atrophic myometrium, histologically proven FIGO stage IA; (c) schematic diagram.

In our study, subjective evaluation was confirmed as the most reliable method to assess myometrial invasion. The possible reason for the superiority of subjective assessment of myometrial invasion is that it can take more features into account other than size and proportion, including dynamic tests (e.g., sliding sign of tumor against uterine wall or in endocervical canal) or vascular pattern [42]. Furthermore, every objective model requires a subjective identification of endometrial tumor and determination of its borders. Although both US objective calculations, deepest invasion/normal myometrium width (Gordon's) ratio and tumor/uterine AP diameter (Karlsson's) ratio, had similar accuracy, the approach published by Gordon et al. performed better in preoperative staging of large polypoid tumors, filling uterine cavity and compressing the already thin postmenopausal myometrium (Figure 3). Such tumors often cause the overestimation of myometrial invasion by Karlsson's model and even by subjective assessment as was shown in our previous paper [43]. On the other hand, in our opinion Gordon's model might be more difficult to reproduce.

Our findings were compared to the results of the meta-analysis published by Alcázar et al. in 2015. The authors did not observe significant differences amongst the three approaches in terms of diagnostic performance but on very heterogeneous data [19]. Moreover, no study included in the meta-analysis used all three methods in one study cohort. Multicenter prospective study by Mascilini et al. investigated several objective ultrasound markers to predict the deep myometrial invasion in the same set of 144 patients [12]. Amongst the objective models, tumor/uterine AP (Karlsson's) ratio at cut-off 0.53 had the best diagnostic performance comparable to the accuracy of subjective evaluation (sensitivity of 72% versus 77% and specificity of 76% versus 81%). However, two recent papers showed significantly lower sensitivity of Karlsson's model in comparison with subjective assessment, both in subgroup of G1 or G2 endometrioid carcinomas (32% versus 80% and 47% versus 73%, resp.) [14, 16]. The explanation might be high prevalence of rather small endometrial tumors with tumor/uterine AP (Karlsson's) ratio < 0.5. Such tumors might invade deeply to the myometrium causing false negative results on preoperative US.

Although we showed higher overall accuracy of subjective assessment of cervical invasion than of myometrial invasion, sensitivity of cervical invasion was significantly lower. In detailed analysis, the low sensitivity was predominantly associated with cases where cervical invasion occurred without deep myometrial invasion (14 out of 22 false negative US findings, 63.6%). The pathological evaluation of these specimens revealed only microscopic infiltration of cervical stroma. These cases were underestimated on US and if excluded, the sensitivity would improve apparently reaching 52.2% (95% CI, 30.6–73.2%). Basically, microscopic cervical stromal infiltration represents diagnostic difficulty for all imaging modalities.

The recently concluded large prospective multicenter study (2011–2015, International Endometrial Tumor Analysis, IETA 4) on 1714 consecutive women with endometrial cancer will be aimed at development and validation of new objective models potentially improving the diagnostic accuracy of ultrasound in preoperative endometrial cancer staging. In addition, within this study the inter- and intraobserver variability in endometrial cancer staging will be tested to document the reproducibility of subjective assessment.

## 5. Conclusions

In conclusion, predictive power of ultrasound based on subjective evaluation by expert was efficient in preoperative local staging of endometrial cancer and sensitivity of subjective assessment of myometrial invasion was superior to investigated objective models.

## Figures and Tables

**Table 1:** Demographic and clinical characteristics of 210 women with histologically confirmed endometrial cancer.

Characteristic	Value
Age (years)	66 (53; 83)
Body mass index (kg/m^2^)	30 (21; 47)
Postmenopausal	193 (91.9)
Family history of breast and/or gynecological cancer	23 (10.9)
Current high/medium potency hormone use^1^	12 (5.7)
Current low potency estrogen use^2^	1 (0.5)
Current tamoxifen use	2 (0.9)
*FIGO stage* ^*3*^	
IA	108 (51.4)
IB	56 (26.7)
II	18 (8.6)
IIIA	6 (2.9)
IIIB	0 (0.0)
IIIC1	8 (3.8)
IIIC2	8 (3.8)
IVA	0 (0.0)
IVB	6 (2.9)
*Histological subtype and grade*	
Endometrioid adenocarcinoma, Grade 1	100 (47.6)
Endometrioid adenocarcinoma, Grade 2	59 (28.1)
Endometrioid adenocarcinoma, Grade 3	26 (12.4)
Nonendometrioid adenocarcinoma	25 (11.9)

Data are given as median (5th percentile; 95th percentile) for continuous variables; *n* (%) for categorical variables. ^1^Oral/dermal estradiol in combination with sequential or continuous progesterone. ^2^Oral/vaginal estriol or vaginal estradiol. ^3^International Federation of Gynecology and Obstetrics (FIGO) 2009 staging criteria.

**Table 2:** Ultrasound parameters of tumors in 210 women with histologically confirmed endometrial cancer.

Characteristic	Value
*Image quality*	
Good	119 (56.7)
Moderate	54 (25.7)
Poor	37 (17.6)
*Uterine size (mm)*	
Craniocaudal	71 (53; 106)
Anteroposterior	40 (28; 58)
Laterolateral	52 (36; 77)
*Tumor size (mm)*	
Craniocaudal	29 (11; 58)
Anteroposterior	16 (5; 42)
Laterolateral	29 (8; 56)
*Objective parameters of myometrial invasion (mm)*	
Deepest myometrial invasion width	7 (0; 15)
Minimal tumor-free margin	6 (0; 17)
Normal myometrium width	12 (5; 20)
*Subjective assessment of tumor invasion*	
Deep myometrial invasion (≥50%)	102 (48.6)
Present cervical stromal invasion	25 (11.9)
*Uterine fibroids*	
Absent	142 (67.6)
Present	68 (32.4)

Data are given as median (5th percentile; 95th percentile) for continuous variables; *n* (%) for categorical variables.

**Table 3:** Diagnostic performance of ultrasound in local staging of endometrial cancer in relation to final histological results as reference standard: subjective assessment and objective methods.

Assessment of myometrial invasion	Sensitivity (95% CI)	Specificity (95% CI)	PPV (95% CI)	NPV (95% CI)	Accuracy (95% CI)	AUC (95% CI)	*p* value
(1) Subjective evaluation of myometrial invasion	79.3 (69.3–87.3)	73.2 (64.4–80.8)	67.6 (57.7–76.6)	83.3 (74.9–89.9)	75.7 (69.3–81.4)	0.712 (0.649–0.768)	<0.001
(2) Deepest invasion/normal myometrium width	69.6 (58.2–79.5)	65.9 (56.8–74.2)	56.7 (46.3–66.7)	77.1 (67.9–84.8)	67.3 (60.3–73.5)	0.677 (0.608–0.741)	<0.001
(3) Tumor/uterine AP diameter	56.3 (45.3–66.9)	76.4 (67.9–83.6)	62.8 (51.1–73.6)	71.2 (62.7–78.8)	68.1 (61.3–74.3)	0.664 (0.595–0.727)	<0.001
*Assessment of cervical stromal invasion*							
(4) Subjective evaluation of cervical stromal invasion	40.5 (24.8–57.9)	94.2 (89.6–97.2)	60.0 (38.2–79.2)	88.1 (82.5–92.4)	84.8 (79.2–89.3)	0.694 (0.626–0.757)	<0.001
*Statistical test*							
Statistical comparison (1) versus (2) (*p* value)^*∗*^	0.023	0.105	—	—	0.047	0.437	
Statistical comparison (1) versus (3) (*p* value)^*∗*^	<0.001	0.651	—	—	0.084	0.309	
Statistical comparison (1) versus (4) (*p* value)^*∗*^	<0.001	<0.001	—	—	0.019	0.687	

^*∗*^Comparison of predictive power of subjective evaluation versus objective methods in assessment of myometrial invasion and comparison of subjective evaluation of myometrial and cervical stromal invasion (chi-square test); estimates of NPV and PPV were not compared due to their dependence on the prevalence of endpoint; NPV, negative predictive value; PPV, positive predictive value; AUC, area under the curve (Receiver Operating Characteristics curves) with corresponding confidence interval and statistical significance (*p* value).

**Table 4:** Characteristics and results of the studies assessing diagnostic performance of ultrasound in preoperative local staging of endometrial cancer.

Reference	Consecutive recruitment	*n*	Myometrial invasion							Cervical stromal invasion		
			Subjective assessment		Karlsson's ratio		Gordon's ratio		Cases with MI ≥ 50% (*n*)	Subjective assessment		Cases with cervical stromal invasion (*n*)
			Sensitivity	Specificity	Sensitivity	Specificity	Sensitivity	Specificity		Sensitivity	Specificity	
Weber (1995) [20]	Unclear	80	—	—	90.0	85.5	—	—	27	—	—	—
Gabrielli (1996) [21]	Unclear	67	—	—	88.0	71.0	—	—	26	54.0	87.0	11
Olaya (1998) [22]	Yes	50	—	—	94.1	84.8	—	—	17	—	—	—
Alcázar (1999) [23]	Yes	50	—	—	86.7	94.3	—	—	15	—	—	—
Arko (2000) [24]	Unclear	120	—	—	—	—	79.0	69.0	48	—	—	—
Fishman (2000) [25]	Yes	91	—	—	87.8	82.7	—	—	33	—	—	—
Szánthó (2001) [26]	Unclear	52	—	—	86.0	90.0	—	—	28	—	—	—
Van Doorn (2002) [27]	Unclear	93	79.0	72.0	—	—	—	—	33	—	—	—
Sawicki (2003) [11]	Unclear	90	—	—	88.9	92.6	—	—	36	86.4	85.3	22
De Smet (2006) [15]	Yes	97	61.0	86.0	72.0	71.0	—	—	59	—	—	—
Takač (2007) [28]	Unclear	53	—	—	—	—	85.7	76.0	28	—	—	—
Yahata (2007) [29]	Unclear	177	—	—	—	—	64.3	97.5	58	—	—	—
Savelli (2008) [7]	Yes	74	84.0	83.0	—	—	—	—	32	93.0	92.0	12
Alcázar (2009) [13]	Yes	96	92.6	82.3	—	—	—	—	27	—	—	—
Ӧzdemir (2009) [30]	Unclear	64	85.0	75.0	—	—	—	—	20	—	—	—
Akbayir (2011) [10]	Yes	298	—	—	68.4	82.0	—	—	98	76.5	99.3	17
Akbayir (2012) [31]	Yes	219	62.0	81.0	—	—	—	—	69	—	—	—
Savelli (2012) [32]	Unclear	155	75.0	89.0	—	—	—	—	76	—	—	—
Ørtoft (2013) [33]	Yes	156	—	—	—	—	77.0	72.0	66	38.0	89.0	26
Mascilini (2013) [12]	Yes	144	77.0	81.0	72.0	76.0	—	—	60	54.0	93.0	26
Antonsen (2013) [6]	Yes	318	71.0	72.0	—	—	—	—	82	29.0	92.0	63
Van Holsbeke (2014) [16]	Unclear	211	83.0	71.0	66.0	74.0	—	—	77	—	—	—
Alcázar (2015) [14]	Yes	169	79.5	89.6	31.8	94.3	—	—	44	—	—	—
Christensen (2015) [34]	Yes	110	—	—	—	—	62.0	83.0	47	25.0	90.0	18
Frühauf (2017)	Yes	210	79.3	73.2	56.3	76.4	69.6	65.9	87	40.5	94.2	37

In all studies, the index test was transvaginal ultrasound and reference standard was final histological finding after hysterectomy.
